# Oximetry Signal Processing Identifies REM Sleep-Related Vulnerability Trait in Asthmatic Children

**DOI:** 10.1155/2013/406157

**Published:** 2013-10-30

**Authors:** Geovanny F. Perez, Maria J. Gutierrez, Shehlanoor Huseni, Khrisna Pancham, Carlos E. Rodriguez-Martinez, Cesar L. Nino, Gustavo Nino

**Affiliations:** ^1^Division of Pulmonary and Sleep Medicine, Children's National Medical Center, Washington, DC 20010, USA; ^2^Division of Pediatric Rheumatology, Allergy & Immunology, Pennsylvania State University College of Medicine, Hershey, PA 17033, USA; ^3^Department of Pediatrics, School of Medicine, Universidad Nacional de Colombia, Bogota, Colombia; ^4^Department of Pediatric Pulmonology and Pediatric Critical Care Medicine, School of Medicine, Universidad El Bosque, Bogota, Colombia; ^5^Research Unit, Military Hospital of Colombia, Bogota, Colombia; ^6^Department of Electronics Engineering, Javeriana University, Bogota, Colombia; ^7^Department of Integrative Systems Biology and Center for Genetic Medicine Research, Children's National Medical Center, George Washington University, Washington, DC 20010, USA

## Abstract

*Rationale*. The sleep-related factors that modulate the nocturnal worsening of asthma in children are poorly understood. This study addressed the hypothesis that asthmatic children have a REM sleep-related vulnerability trait that is independent of OSA. *Methods*. We conducted a retrospective cross-sectional analysis of pulse-oximetry signals obtained during REM and NREM sleep in control and asthmatic children (*n* = 134). Asthma classification was based on preestablished clinical criteria. Multivariate linear regression model was built to control for potential confounders (significance level *P* ≤ 0.05). *Results*. Our data demonstrated that (1) baseline nocturnal respiratory parameters were not significantly different in asthmatic versus control children, (2) the maximal % of SaO_2_ desaturation during REM, but not during NREM, was significantly higher in asthmatic children, and (3) multivariate analysis revealed that the association between asthma and REM-related maximal % SaO_2_ desaturation was independent of demographic variables. *Conclusion*. These results demonstrate that children with asthma have a REM-related vulnerability trait that impacts oxygenation independently of OSA. Further research is needed to delineate the REM sleep neurobiological mechanisms that modulate the phenotypical expression of nocturnal asthma in children.

## 1. Introduction

 Asthma is a chronic inflammatory disease characterized by worsening of symptoms during sleep [1, 2]. This nocturnal vulnerability of asthmatic individuals has been previously attributed to increased vagal tone, decreased sympathetic activity, reduced functional residual capacity of the lungs (affecting the small airways), endogenous circadian system changes during the night, and a higher risk for obstructive sleep apnea (OSA) in the asthmatic population [2–4]. Interestingly, several studies have reported that asthmatic patients deteriorate more during the morning hours with the majority of respiratory arrests and sudden deaths occurring from midnight to 8 am [5, 6]. The latter phenomenon suggests a greater influence of the rapid eye movement (REM) sleep in the asthmatic condition since this sleep stage predominates during the second part of the night [7].

REM sleep is considered particularly important in the pathogenesis of OSA and other sleep-related breathing abnormalities [8–12]. It has also been suggested that REM sleep is characterized by nocturnal bronchoconstriction [13]. In the context of pediatric asthma, we have recently identified that asthmatic children with OSA have more REM sleep-related breathing abnormalities relative to children with OSA alone [14]. These findings suggested that asthmatic patients have a REM sleep vulnerability trait that predisposes them to have more respiratory abnormalities during this sleep stage. In support of this notion, Catterall et al. have previously demonstrated that stable adult asthmatics have irregular breathing and hypoxemia clustered during REM sleep [15]. Importantly, OSA worsens during REM sleep as well [8–12], thus REM-related breathing abnormalities in asthmatic individuals may be just a reflection of the well-known association between asthma and OSA [4]. Accordingly, to better delineate the link between asthma and REM sleep in children, our current study investigated whether asthmatic children have more REM sleep-related hypoxemia independently of the presence of OSA. To this end, we conducted stage specific (REM versus NREM sleep) signal processing of the pulse-oximetry channel recorded during overnight polysomnography (PSG) in asthmatic children without OSA as previously described [14]. Our main hypothesis was that asthmatic children without OSA would have more REM-related hypoxemia, expressed as the maximal percentage of SaO_2_ desaturation during REM sleep, relative to control children without asthma. Secondary analysis evaluated the influence of modulatory factors of REM-related breathing abnormalities, such as gender, age, and ethnicity [16], in the association between REM-related hypoxemia and asthma in children.

## 2. Materials and Methods

### 2.1. Subjects

A database of all children that underwent routine overnight polysomnogram (PSG) at the Penn State Sleep Research and Treatment Center (SRTC) between January 2010 and June 2011 was reviewed. Patients were eligible for the study if their PSG ruled out the diagnosis of obstructive sleep apnea (OSA). Almost all patients were referred to PSG to rule out OSA. OSA was defined as an obstructive apnea-hypopnea index (OAHI) ≥ 1.5 events per hour [17–20]. Only the patient's initial PSG was included in the study. Infants, school age children, and young adolescents were included (2–13 years). Patients were excluded if they had need for supplemental oxygen or positive airway pressure, central hypoventilation syndromes, congenital heart disease, severe developmental delay, cerebral palsy, genetic syndromes, craniofacial abnormalities, or neuromuscular disorders. Patients without complete clinical and PSG data available were also excluded from the study. Asthma status was determined based on electronic medical records reviewed in Penn State Children's Hospital and Penn State SRTC. This study was approved by the Institutional Review Board of Penn State College of Medicine.

### 2.2. PSG Protocol, Scoring, and Analysis

Standard pediatric overnight PSG was performed on all patients. During 9-10 hours, the child's sleep was continuously recorded to a computerized system (Twin PSG software; Grass Technologies. Inc., West Warwick, RI, USA) and scored manually in 30-second epochs according to the American Academy of Sleep Medicine (AASM) standardized criteria [17]. PSG measurements included electroencephalograms (EEG), electrooculograms (EOG), electrocardiogram (ECG), mental-submental electromyogram (EMG), thoracic and abdominal wall motion (respiratory inductance plethysmography), pulse oximetry (with 2 s averaging time), end-tidal carbon dioxide monitoring, combined nasal/oral thermistor, and nasal pressure. Sleep stages and respiratory events were scored according to the AASM pediatric scoring criteria [17]. Five AASM sleep stages were identified (wake stage = W, stage 1 = N1, stage 2 = N2, stage 3 = N3, and stage REM = R). For the purpose of this investigation, we used OAHI and respiratory data calculated for W stage (beginning of PSG), non-REM stage (represented by N1, N2, and N3), and REM sleep. The OAHI was calculated separately during REM, NREM sleep, and total sleep time (TST). Pulse-oximetry signal was examined separately and carefully cleaned from artifacts. “Maximal percentage of SaO_2_ desaturation” was calculated with the formula (SaO_2_ baseline − SaO_2_ nadir)/SaO_2_ baseline, and it was individually measured during NREM and REM sleep stages as previously described [14]. Additional respiratory analyses were conducted to obtain baseline SaO_2_ during TST, wake, NREM, and REM sleep.

### 2.3. Asthma Status and Other Covariables

Clinical and demographic variables were obtained reviewing electronic medical records in Penn State Children's Hospital and Penn State Sleep Research and Treatment Center. “Asthma” was defined in this pediatric population using a definition that required the presence of at least one of the following criteria: (1) ever being diagnosed with asthma by a physician on the basis of criteria recommended for children 0–4 and 5–11 years of age in the National Institute Of Health (NIH) National Asthma Education and Prevention Program (NAEPP) Guidelines [1] and (2) use of asthma therapy and/or presence of asthma symptoms in the past 12 months. Other covariables investigated included age, race, sex, body mass index (BMI), percent of REM sleep, total sleep time (TST), and TST in supine position.

### 2.4. Statistical Analysis

Data were analyzed using the software SAS version 9.3 or later (SAS Institute Inc., Cary, NC, USA). Exploratory data analysis on main variables were performed for the entire study population and stratified according to asthma status. SaO_2_ parameters were calculated separately for REM and NREM sleep. For pair-wise relationships, two-sample *t*-test was used to compare the mean value of the continuous outcome measures such as SaO_2_-derived measurements, and chi-square test was used to compare the proportion of positive signals for binary outcomes. Multivariate linear regression model was built to study the joint effect of asthma and maximal percentage of SaO_2_ desaturation during REM with control of possible confounders such as gender, age, and ethnicity. Significance was taken at the *P* < 0.05 level.

## 3. Results

### 3.1. Study Population

134 children and young adolescents (2–13 years) were included in this study. The total study population was subdivided into one group with asthma (*n* = 46) and another group without the disease (*n* = 88). Comparison of demographic, anthropometric, and baseline PSG variables between these two groups revealed no significant differences (Tables 1 and 2).

### 3.2. Children with Asthma Are Susceptible to Nocturnal Hypoxemia during REM Sleep

**Figure 1:**
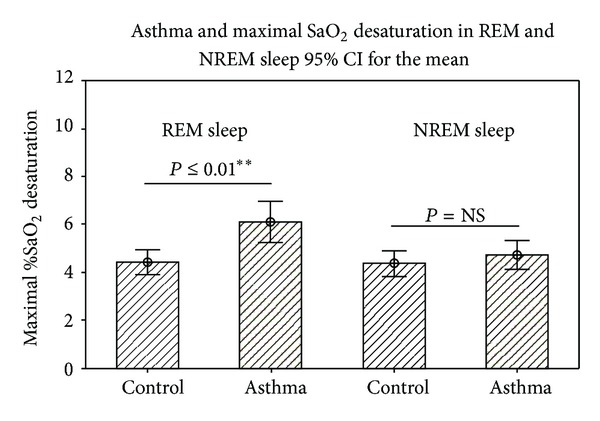
Maximal SaO_2_ desaturation in REM and NREM sleep by asthma status in children. Data are presented as mean and 95% confidence interval (CI). REM: rapid eye movement; NREM: nonrapid eye movement; SaO_2_: Oxygen saturation; *P* values are obtained by two-sample student *t*-test.

We first examined pulse-oximetry signals at baseline and during sleep stages (REM versus NREM) in children with asthma and contrasted them with those seen in children without this condition (control). As shown in Table 2, the mean SaO_2_ parameters obtained during wake, REM, and NREM sleep were not significantly different in asthmatic children compared to those in the control group (Table 2). In the same way, the maximal percentage of SaO_2_ during REM and NREM was not significantly different in those children without asthma (REM 4.4% ± SD 2.4 versus NREM 4.2% ± SD 2.5, *P* = 0.38) (Figure 1). The mean SaO_2_ nadir in the asthma group was slightly lower (93.49 ± SD 2.2) relative to the control group (94.14 ± SD 1.5), but this difference did not reach statistically significance (*P* = 0.08) probably because this nadir included REM and NREM sleep values. In contrast, children with asthma had a maximal percentage of SaO_2_ desaturation that was significantly higher during REM relative to that seen in NREM (REM 6.2% ± SD 2.9 versus NREM 4.4 ± SD 2.0, *P* = 0.001). 

### 3.3. The Association between Asthma and REM-Related Hypoxemia Is Independent of Gender, Age, and Ethnicity

REM-related breathing abnormalities have been linked to younger age and female gender [16]. Accordingly, we built a multivariate linear regression model to assess the confounder effect of age, gender, and ethnicity in the relationship between asthma and maximal percentage of SaO_2_ desaturation during REM sleep (Table 3). After adjusting for these covariables, we found that the effect of asthma in REM-related hypoxemia (maximal % REM SaO_2_ desaturation) is independent of age, gender, and ethnicity (adjusted *P* = 0.04, Table 3). 

## 4. Discussion

The most important finding of the current study is that the maximal percentage of SaO_2_ desaturation during REM, but not during NREM, is significantly greater in asthmatic children without OSA. Accordingly, our data suggest that children with asthma have a REM-related vulnerability trait that impacts oxygenation independently of OSA according to PSG criteria. 

There is clear evidence supporting the worsening of asthma during sleep. The largest study of the prevalence of nocturnal asthma symptoms was reported by Turner-Warwick [21]. This survey of 7729 patients with asthma revealed that 74% awoke at least once per week with asthma symptoms [21]. Several asthma studies have also shown decreased pulmonary function and increased inflammatory markers at nighttime. Kelly et al. [22] demonstrated that the forced expiratory volume in the first second (FEV1) of patients with asthma is significantly worse at 4:00 A.M. compared to 4 P.M. [22]. Bonnet et al. performed inhalation challenges every 4 h for 13 consecutive times in asthmatic patients and found 24 hour oscillations in the pulmonary sensitivity to histamine and methacholine, with at least doubling concentrations required for the same effect at certain times of day [23]. Additionally, Kraft et al. [24] reported that patients with nocturnal asthma exhibit higher concentrations of inflammatory markers in the distal airways (leukocyte, neutrophil, and eosinophil counts) at nighttime [24]. Collectively, these data support the prevailing notion that the asthmatic condition is highly influenced by the nocturnal phase of circadian rhythms.

In concert with circadian changes at night, specific sleep stages appear to modulate the phenotypical expression of asthma. Shapiro et al. reported that nocturnal bronchoconstriction may be associated with REM sleep [13]. Catterall et al. also identified that individuals with chronic stable asthma have irregular breathing and greater fluctuations in SaO_2_ during REM sleep relative to controls [15]. In children, we have recently reported that asthmatic subjects with OSA have clustering of obstructive events and hypoxemia during REM sleep compared to children with OSA alone [14]. Based on these observations, our current study postulated that asthmatic children without OSA also have nocturnal respiratory abnormalities during REM sleep. To test this hypothesis, we conducted signal processing of the pulse-oximetry channel used during PSG to calculate stage specific (REM versus NREM) means and maximal SaO_2_ desaturations as previously described [14]. Our data demonstrated that children with asthma have greater REM-related maximal SaO_2_ desaturation relative to those children without asthma. 

There are several neurobiological mechanisms that might underlie the potential modulatory role of REM in asthma. REM sleep is characterized by increased cholinergic outflow and ablated noradrenergic signals in the brainstem [25–27], which are in turn critical modulators of the caliber and reactivity of the lower airways in mice [25–27]. In addition, animal models of allergic asthma have illustrated that allergic lung inflammation may affect central noradrenergic control of cholinergic outflow to the airways, thereby augmenting bronchoconstrictive reflex responses in the asthmatic state [28]. The tone of the accessory respiratory muscles is also markedly decreased during REM sleep, which may reduce pulmonary reserve and lead to hypoventilation-hypoxemia in many respiratory conditions including asthma [29]. The latter information, together with our current findings, support the notion that REM sleep neurobiology plays a key role in the normal functioning of the lower airways and so it might modulate the nocturnal manifestations of asthma children.

In evaluating the implications of the present observations, it is important to mention that our investigation used a cross-sectional design with a retrospective cohort of children. Thus, this study has the potential limitations of any retrospective analysis such as selection bias and misclassification of disease (information bias) resulting from inaccurate documentation in medical records. In terms of potential confounders, our design and statistical analysis accounted for several important covariables for the association between REM-related hypoxemia and asthma. For instance, our study excluded children with chronic lung disease, neuromuscular disorders, cerebral palsy, craniofacial abnormalities, and dysmorphic genetic syndromes, as they have a higher risk for nocturnal hypoxemia. Nonetheless, snoring was not evaluated and was likely present in a large number of children included in the study, so they cannot be necessarily regarded as otherwise healthy children with asthma. On the other hand, the strengths of our study included the relatively large sample size and the use of objective data to evaluate the influence of REM in asthma (PSG scoring and sleep-stage signal processing).

In summary, we feel that enough evidence is presented to support the concept that children with asthma have a REM-related vulnerability trait that impacts oxygenation independently of OSA. Further research is needed to delineate the biological link between REM-sleep and the physiology of the lower airways during health and disease. New knowledge in this area may allow the discovery of mechanistic pathways that connect airway inflammation and function with sleep biology, which in turn may result in novel strategies for the treatment of nocturnal symptoms of asthma.

## Figures and Tables

**Table 1:** Demographic profile of subjects.

Factors/variables	Total (*n* = 134)	Control (*n* = 88, 66%)	Asthma (*n* = 46, 34%)	*P* value
*Demographic variables *				
Gender				0.28
Female	58 (43%)	41 (46%)	17 (37%)	
Male	76 (57%)	47 (54%)	29 (63%)	
Age: mean (SD)	6.32 (2.9)	6.1 (2.9)	6.8 (2.9)	0.18
Ethnicity				0.95
White	96 (62%)	55 (67%)	41 (65%)	
Others	49 (37%)	27 (33%)	22 (35%)	
BMI: mean (SD)	18.5 (5.3)	17.8 (4.3)	19.6 (5.9)	0.08

For quantitative variables, data are presented as mean ± standard deviation (SD). BMI: body mass index; for categorical variables, data are presented as count number (column percentage). *P* values are obtained by either two-sample *t*-test or chi-square test, depending on the type of variables.

**Table 2:** Polysomnographic parameters of subjects.

Factors/variables	Control (*n* = 88, 66%)	Asthma (*n* = 46, 34%)	*P* value
*Sleep study parameters *			
OAHI			
Events/hr; mean (SD)	0.35 (0.4)	0.39 (0.4)	0.65
Total sleep time (TST)			
Min; mean (SD)	535 (61)	526 (41)	0.28
TST supine %			
Mean (SD)	43.3 (27.2)	46.3 (22)	0.67
REM (%)			
Mean (SD)	18.8 (5.1)	21.3 (4.9)	0.35
Max % REM SAO_2_ desaturation			
Mean (SD)	**4.4 (2.5)**	**6.2 (2.8)**	0.001**
Max % NREM SAO_2_ desaturation			
Mean (SD)	4. 4 (2.5)	4.7 (2.0)	0.38
SAO_2_ (wake)			
Mean (SD)	97.3 (1.4)	97.2 (0.9)	0.54
SAO_2_ (REM, average)			
Mean (SD)	97 (1.0)	97 (1.2)	0.85
SAO_2_ (NREM, average)			
Mean (SD)	96.8 (1.0)	96.6 (1.2)	0.28

For quantitative variables, data are presented as mean ± standard deviation (SD). SAO_2_: saturation of oxygen; REM: rapid eye movement; OAHI: obstructive apnea-hypopnea index. For categorical variables, data are presented as count number (column percentage). *P* values are obtained by either two-sample *t*-test or chi-square test, depending on the type of variables.

Statistically significant results are shown in bold font.

**Table 3:** Multivariate regression analysis results for the association between rhinitis and REM-OAHI adjusted by co-variables.

Multivariate regression analysis for max % REM SAO_2_ desaturation			
Predictors	Parameter estimate	SE	*P*-value
Asthma	**1.26**	**0.62**	0.04*
Age yrs	−0.08	0.10	0.41
Gender	−0.90	0.59	0.13
Ethnicity	−0.18	0.27	0.50

Data are presented as parameter estimate, standard error (SE). REM: rapid eye movement; OAHI: obstructive apnea-hypopnea index. Adjusted *P*-values are obtained by multiple linear regression.

Statistically significant results are shown in bold font.
